# Apocynin improves endothelial function and prevents the development of hypertension in fructose fed rat

**DOI:** 10.4103/0253-7613.58508

**Published:** 2009-10

**Authors:** Banappa S. Unger, Basangouda M. Patil

**Affiliations:** Department of Pharmacology and Toxicology, KLES's College of Pharmacy, J N Medical College Campus, Nehru Nagar, Belgaum - 590 010, India; 1 KLES's College of Pharmacy, Vidya nagar, Hubli - 580 031, Karnataka, India

**Keywords:** Superoxide, nitric oxide, NAD(P)H oxidase, apocynin, endothelial dysfunction, hypertension

## Abstract

**Background and Objectives:**

Exaggerated production of superoxide and inactivation of nitric oxide have been implicated in pathogenesis of hypertension. NAD(P)H oxidase is one of the major source of reactive oxygen species in vasculature. In the present study, we aimed to determine the effect of chronic administration of Apocynin an NAD(P)H oxidase inhibitor on endothelial function and hypertension in fructose-fed rat.

**Materials and Methods:**

Endothelial function, vascular superoxide, and nitric oxide production/bioavailability in aortas from fructose-fed rats and age-matched controls treated with or without apocynin were assessed using isometric tension studies in organ chambers. Systolic blood pressure was measured by the tail cuff method.

**Results:**

In fructose-fed rats, acetylcholine-induced relaxation was impaired, vascular superoxide production was increased, and nitric oxide bioavailability was decreased along with an increase in systolic blood pressure compared to controls. Apocynin treatment prevented the increased generation of superoxide, decreased nitric oxide bioavailability, impaired acetylcholine-induced relaxation, and elevation of systolic blood pressure.

**Conclusion:**

Chronic administration of apocynin improves the endothelial function by reducing oxidative stress, improving NO bioavailability, and prevents the development hypertension in fructose-fed rat.

## Introduction

Hypertension, a component of metabolic/insulin resistance syndrome, is an important risk factor for cardiovascular disease contributing to the increased morbidity and mortality. Oxidative stress has emerged as an important pathogenic factor in the development of hypertension.[1–5] Exaggerated production of superoxide (O_2_^−^) by the vascular wall has been observed in different animal models of hypertension including fructose-fed rat.[6–9] Growing evidence supports the possibility that increased oxidative inactivation of nitric oxide (NO) by an excess superoxide may account for the decrease in availability of nitric oxide and endothelial dysfunction contributing to elevation of blood pressure.[10,11] A fructose-fed rat, an animal model of insulin resistance state has been shown to exhibit hypertension and endothelial dysfunction.[12,13] Recently, increased superoxide production in vascular tissue, mediated through NAD(P) H oxidase has been noted.[9] We therefore postulate that increased vascular superoxide production by NAD(P)H oxidase is responsible for endothelial dysfunction, which in turn may contribute to the development of hypertension in insulin-resistance state. Hence, in the present study, we aimed to determine the effect of chronic administration of apocynin, an NAD(P)H oxidase inhibitor, on endothelial function and development of hypertension in fructose-fed rats.

## Materials and Methods

### Animals and experimental design

Male SD rats (175-200 g) were procured from National Center for Lab Animal Sciences, National Institute of Nutrition, Hyderabad, India. They were acclimatized for laboratory conditions for 7 days and randomly divided into four groups each having seven animals. (1) Control (C): Fed with standard chow diet; (2) fructose fed (F): Fed with high fructose diet (60% of fructose); (3) control + apocynin (CA): Fed with standard chow diet plus apocynin (1.5 mM) in drinking water; (4) fructose + apocynin (FA): Fed with high fructose diet plus apocynin (1.5 mM) in drinking water. The animals were housed under standard laboratory conditions and maintained under a 12-h light-dark cycle and had free access to drinking water and diet for 8 weeks. The experiment was carried out according to guidelines of Committee for the Purpose of Control and Supervision of Experiments on Animals (CPCSEA), Ministry of Social Justice and Empowerment, Govt. of India. Institutional Animal Ethical Committee approved all the procedures. Systolic blood pressure was measured once in a week. At the end of the experimental period, the rats were killed and thoracic aorta was isolated for isometric tension studies in the organ chamber.

### Measurement of systolic blood pressure

Systolic blood pressure was measured indirectly in a conscious, pre-warmed, and slightly restrained rat by the tail cuff method (Harvard rat-tail blood pressure monitor, USA). An average of eight consecutive readings was noted. For these measurements, rats were trained adequately before the study.

### Isometric tension studies in organ chamber

Aortic ring preparation

Rats were killed by cervical dislocation under mild ether anesthesia and thoracic aorta was isolated and cut into ring segments of approximately 4 mm length and mounted in organ chambers for isometric tension recording. The vessel rings were mounted onto two parallel stainless-steel pins through the lumen and placed in a chamber containing a 20 ml Krebs-Henseleit buffer (pH 7.4) solution, which was maintained at 37°C and gassed with 5% CO_2_ and 95% O_2_. Over a period of 1 h equilibration period, the resting tension was gradually increased to 2 g. The vessels were left at this resting tension throughout the remainder of the study. Isometric tension developed in vasculature was recorded by using a force transducer and a computerized data acquisition system (Biopac Systems Inc., CA). Separate aortic ring preparation (n = 7) was used for each of the following experiments such as endothelium-dependent and -independent relaxation (procedure described in the next section), superoxide production (procedure described in section *Measurement of vascular superoxide production*) and NO bioavailability (procedure described in section *Measurement of vascular NO bioavailability*).

### Measurement of vascular reactivity (endothelium-dependent and -independent relaxation)

Concentration-response curves to acetylcholine (endo thelium-dependent vasodilator) and sodium nitroprusside (endothelium-independent vasodilator) were performed after submaximal precontraction of the vessel with phenyephrine (1 × 10^−6^ M). The seven number of aortic ring preparation each isolated from seven animals per group were used for experiment (i.e. N = 7).

### Measurement of vascular superoxide production

A main factor limiting the bioavailability of NO is the superoxide anion radical (O_2_^−^). We therefore assessed O_2_^−^ formation in the aorta. Super oxide level was measured as Tempol (1 × 10^−4^ M, SOD mimetic) induced relaxation of isolated rat aorta after submaximal precontraction of the vessel with phenylephrine (1 × 10^−6^ M) as previously described.[14] To prevent synthesis of prostaglandins, we performed the experiments in the presence of 10 mM indomethacin. The seven aortic ring preparations each isolated from seven animals per group were used for experiment (i.e., N = 7).

### Measurement of vascular NO bioavailability

Aortic nitric oxide bioavailability was measured as a NOS inhibitor [NG-nitro-L-arginine methyl ester (L-NAME, 1 × 10^−4^ M)] induced contraction of isolated rat aorta after submaximal precontraction of the vessel with phenylephrine (1 × 10^−6^ M) as previously described.[14] To prevent synthesis of prostaglandins, we performed the experiment in the presence of 10 mM indomethacin. The seven number of aortic ring preparation each isolated from seven animals per group were used for the experiment (i.e. N = 7).

Drugs and chemicals

L-NAME, tempol, acetylcholine, sodium nitroprusside, L-phenyephrine, and apocynin/acetovanillone were purchased from Sigma Chemical Company Inc., St Louis, MO, USA. Indomethacin was obtained as a gift sample from Sun Pharma, Chennai, India. All other chemicals used were analytical grade purchased from Himedia Laboratories Ltd, Mumbai, India.

Data analysis

Data in the manuscript are expressed as Mean ± SEM. Comparisons between groups were made using one-way ANOVA. When significance was indicated, a Student-Newman-Keuls post hoc analysis was used. Statistical significance was assumed at *P* < 0.05.

## Results

### Systolic blood pressure

**Figure 1:**
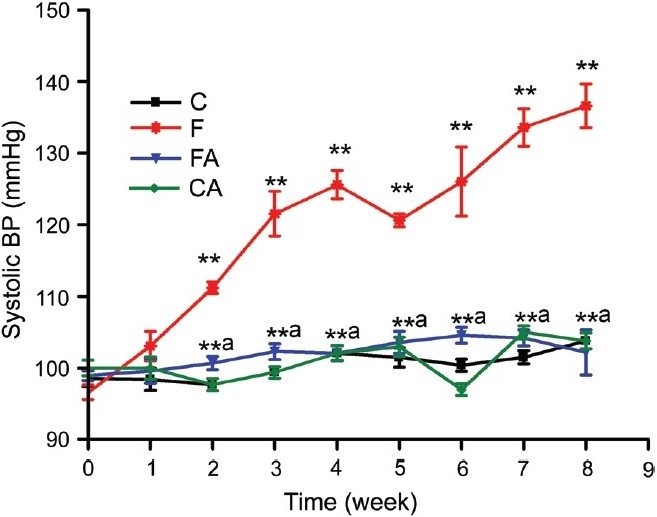
Systolic blood pressure in control and fructose fed-animals treated with or without apocynin. Control, control + apocynin, fructose fed, fructose + apocynin. **P < 0.01 F vs. C, **P < 0.01 FA vs. F

Fructose-fed rats exhibit elevation in systolic blood pressure within 1 week (14.41%, *P* > 0.05) but became significantly high by the second week (18.8% *P* < 0.001) and continued to increase till eighth week (24% *P* < 0.001) compared to chow-fed control [Figure 1]. Interestingly, this increase was completely prevented in apocynin-treated fructose-fed rats [Figure 1]. However, apocynin had no effect on systolic blood pressure in chow-fed control rats.

Vascular reactivity (endothelium-dependent and -independent relaxation)

**Figure 2:**
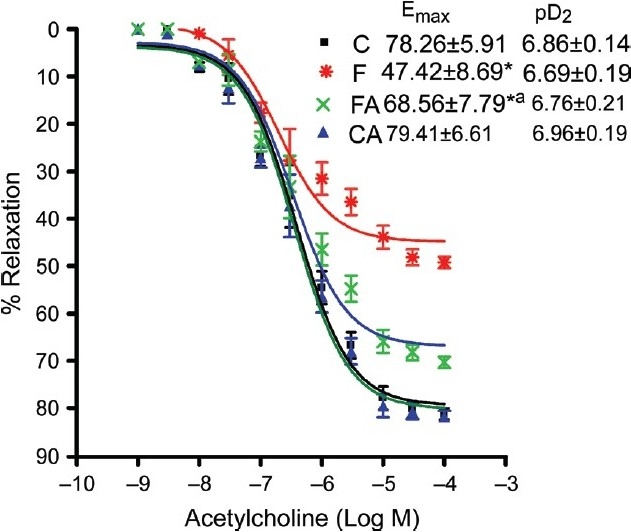
Acetylcholine-induced relaxation of isolated rat aorta preconstricted with phenylephrine in control and fructose-fed animals treated with or without apocynin. Control, control + apocynin, fructose fed, fructose + apocynin. *P < 0.05 F vs. C, *P < 0.05 FA vs. F

**Figure 3:**
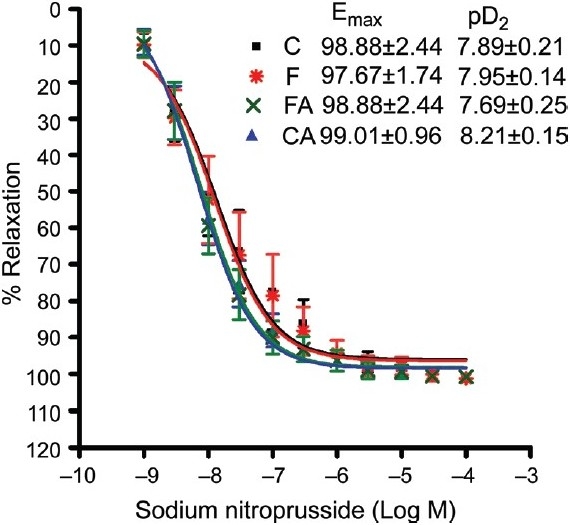
Sodium nitroprusside-induced relaxation of isolated rat aorta preconstricted with phenylephrine in control and fructose-fed animals treated with or without apocynin. Control, control + apocynin, fructose fed, fructose + apocynin

The endothelium-dependent vasodilator acetylcholine (Ach) and endothelium-independent vasodilator (SNP) elicited the concentration-dependent relaxation of phenylephrine preconstricted isolated aortic rings of all animals [Figures 2 and 3]. Aortic rings of fructose-fed rat had a markedly impaired endothelium-dependent relaxation as compared to control [Figure 2]. As shown in Figure 2, the maximum relaxation response (*E*_max_) induced by acetylcholine was significantly reduced in aortas isolated from high fructose diet-fed rats when compared to chow-fed rats (47.42 ± 8.69 vs. 78.26 ± 5.91, *P* < 0.05). Note, this impaired response to Ach was normalized by treatment with the NAD(P)H oxidase inhibitor apocynin (68.56 ± 7.79 vs. 47.42 ± 8.69, *P* < 0.05) [Figure 2]. The sensitivity of the aortae from all experimental groups to acetylcholine was similar as indicated by no significant difference in pD_2_ values among the groups. The sensitivity (pD_2_) and maximum relaxation response (*E*_max_) induced by the endothelium-independent vasodilator sodium nitroprusside were not significantly different among the groups [Figure 3].

### Vascular superoxide production

**Figure 4:**
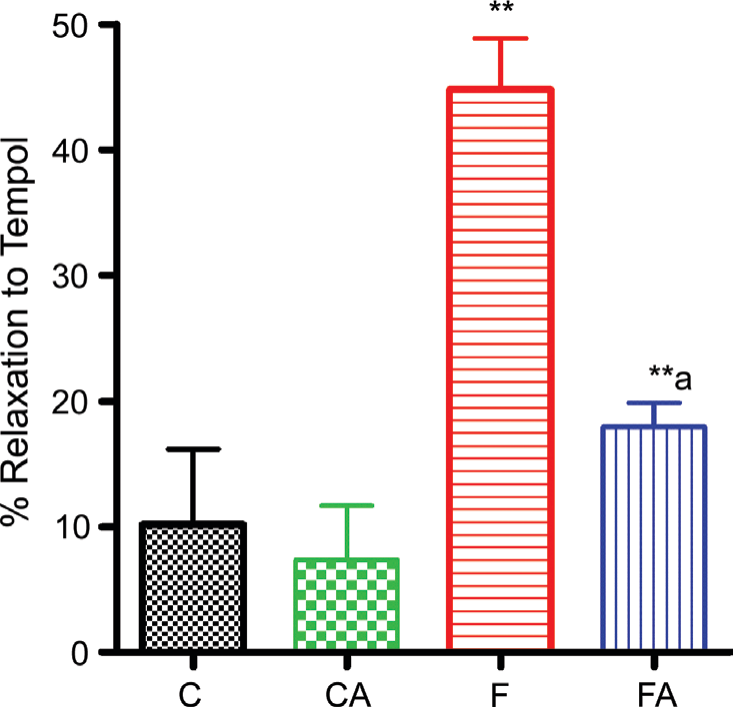
Super-oxide level measured as tempo-induced relaxation of isolated rat aorta preconstricted with phenylephrine in control and fructose-fed animals treated with or without apocynin. Control, control + apocynin, fructose fed, fructose + apocynin. **P < 0.01 F vs. C, **P < 0.01 FA vs. F

A significant increase in tempol (SOD mimetic)-induced relaxation of isolated rat aorta preconstricted with phenylephrine revealed a marked increase in vascular O_2_^−^ in aorta isolated from fructose-fed rats compared with levels in aorta isolated from control. This increase was prevented in aortic rings isolated from apocynin-treated fructose-fed rats [Figure 4]. However, in chow-fed control, apocynin had no significant effect on O_2_^−^ level.

### Vascular NO bioavailability

**Figure 5:**
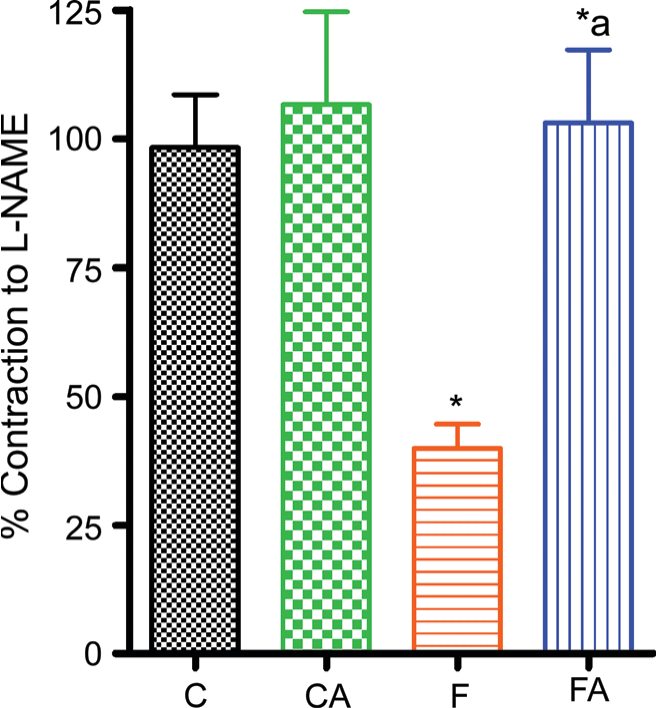
Nitric oxide level measured as L-NAME-induced contraction of isolated rat aorta preconstricted with phenylephrine in control and fructose-fed animals treated with or without apocynin. Control, control + apocynin, fructose fed, fructose + apocynin. *P < 0.05 F vs. C, *P < 0.05 FA vs. F

A significant decrease in L-NAME-induced contraction of isolated rat aorta preconstricted with phenylephrine revealed a marked decrease in vascular NO bioavailability in aorta isolated from fructose-fed rats compared with control. This decrease was prevented in apocynin-treated fructose-fed rat [Figure 5]. However, in chow fed control, apocynin had no significant effect on NO level.

## Discussion and Conclusion

In the present study, we demonstrate for the first time that chronic administration of apocynin, a putative NAD(P)H oxidase inhibitor, prevents the development of endothelial dysfunction and hypertension by inhibiting exaggerated vascular superoxide production and increasing the bioavailability of nitric oxide, supporting the hypothesis that reduced NO bioavailability due to increased reactive oxygen species (ROS, superoxide) plays a critical role in the development of endothelial dysfunction and hypertension in fructose-fed rat.

Growing body of evidence supports the role of ROS in pathogenesis of hypertension. This notion is supported by the observation that vascular ROS production is elevated in different experimental models of hypertension.[6–9] Increased O_2_^−^ level is known to inactivate the vasodilator nitric oxide, leading to endothelial dysfunction, a characteristic feature of many vascular diseases, including hypertension.[15–17] In various animal models of hypertension, an antioxidant superoxide dismutase (SOD) and SOD mimetics were found to lower the blood pressure suggesting that the oxidative inactivation of NO contributes to blood pressure elevation.[18–20] In the recent past, many studies have demonstrated that the major source of ROS in the vasculature is NAD(P)H oxidase. NAD(P)H oxidase enzyme expression and activity are increased in cardiovascular tissues of animal models of hypertension.[21,22]

Apocynin, a methoxy-substituted catechol from the herb *Picrorhiza kurroa*, a orally active reversible inhibitor of NAD(P)H oxidase, has been reported to inhibit the activation of NAD(P)H oxidase by blocking the assembly of a functional NADPH oxidase complex.[23] Administration of apocynin reduces vascular O_2_^−^ production and attenuates hypertension in hypertensive rats.[24–26] Thus, there is compelling evidence to suggest a role for ROS mediated by NAD(P)H oxidase in the pathogenesis of hypertension. Previous studies have demonstrated that apocynin inhibits the NAD(P)H oxidase in the vasculature, indicating suitability, selectivity, and wide usage of this compound as a pharmacological tool for vascular NAD(P)H oxidase inhibition.[23–27]

Fructose-fed rat, a widely used animal model of insulin resistance syndrome, exhibits oxidative stress, endothelial dysfunction, and hypertension. Treatment with antioxidants has been shown to reduce blood pressure in this model.[28,29] Recently, it has been reported that vascular superoxide production is increased in fructose-fed rat, and *in vitro* incubation with apocynin prevents this increase in vascular superoxide production.[9] Further, it has been found that NAD(P)H oxidase expression and activity are increased in cardiovascular tissues in fructose-fed animals.[9,30] However, there was lack of evidence to support contribution of NAD(P)H oxidase-mediated superoxide production to the development of endothelial dysfunction and hypertension in fructose-fed rats. In the present study, we observed that apocynin administration prevented the development of endothelial dysfunction by inhibiting increased generation of superoxide and thus increasing the bioavailability of NO. These observations support the notion that activation of vascular NAD(P)H oxidase plays a critical role in the development of endothelial dysfunction in fructose-fed rats. It has been reported that superoxide production is increased well before (first week) the development of hypertension in aortae from fructose-fed rat and endothelial dysfunction precedes the development of hypertension.[30,31] Thus, it is clearly possible that improved endothelial function as a result of inhibition of NAD(P)H oxidase might contribute to decreased systolic BP in apocynin-supplemented fructose-fed rat.

Fructose-fed rat, a widely used animal model of insulin resistance syndrome, exhibits oxidative stress, endothelial dysfunction, and hypertension. Treatment with antioxidants has been shown to reduce blood pressure in this model.[28,29] Recently, it has been reported that vascular superoxide production is increased in fructose-fed rat, and *in vitro* incubation with apocynin prevents this increase in vascular superoxide production.[9] Further, it has been found that NAD(P)H oxidase expression and activity are increased in cardiovascular tissues in fructose-fed animals.[9,30] However, there was lack of evidence to support contribution of NAD(P)H oxidase-mediated superoxide production to the development of endothelial dysfunction and hypertension in fructose-fed rats. In the present study, we observed that apocynin administration prevented the development of endothelial dysfunction by inhibiting increased generation of superoxide and thus increasing the bioavailability of NO. These observations support the notion that activation of vascular NAD(P)H oxidase plays a critical role in the development of endothelial dysfunction in fructose-fed rats. It has been reported that superoxide production is increased well before (first week) the development of hypertension in aortae from fructose-fed rat and endothelial dysfunction precedes the development of hypertension.[30,31] Thus, it is clearly possible that improved endothelial function as a result of inhibition of NAD(P)H oxidase might contribute to decreased systolic BP in apocynin-supplemented fructose-fed rat.

## Conclusion

This work demonstrates that NAD(P) H oxidase-mediated excessive superoxide production may reduce the bioavailability of NO which may account for endothelial dysfunction and hypertension in fructose-fed rats. These findings may provide additional evidence/basis for therapeutic interventions aimed at reducing superoxide-induced vascular dysfunctions associated with insulin resistance and hypertension.
